# CT Slice Thickness and Convolution Kernel Affect Performance of a Radiomic Model for Predicting EGFR Status in Non-Small Cell Lung Cancer: A Preliminary Study

**DOI:** 10.1038/s41598-018-36421-0

**Published:** 2018-12-17

**Authors:** Yajun Li, Lin Lu, Manjun Xiao, Laurent Dercle, Yue Huang, Zishu Zhang, Lawrence H. Schwartz, Daiqiang Li, Binsheng Zhao

**Affiliations:** 1 Department of Radiology, the Second Xiangya Hospital, Central South University, Changsha, Hunan 410011 China; 2 Department of Radiology, Columbia University Medical Center, New York, NY 10039 USA; 3 Gustave Roussy, Université Paris-Saclay, Inserm, UMR1015 Paris, France; 4 Department of Pathology, the Second Xiangya Hospital, Central South University, Changsha, Hunan 410011 China; 5 Department of Surgery, University of Missouri, Columbia, MO USA

## Abstract

We evaluated whether the optimal selection of CT reconstruction settings enables the construction of a radiomics model to predict epidermal growth factor receptor (EGFR) mutation status in primary lung adenocarcinoma (LAC) using standard of care CT images. Fifty-one patients (EGFR:wildtype = 23:28) with LACs of clinical stage I/II/IIIA were included in the analysis. The LACs were segmented in four conditions, two slice thicknesses (Thin: 1 mm; Thick: 5 mm) and two convolution kernels (Sharp: B70f/B70s; Smooth: B30f/B31f/B31s), which constituted four groups: (1) Thin-Sharp, (2) Thin-Smooth, (3) Thick-Sharp, and (4) Thick-Smooth. Machine learning algorithms selected and combined 1,695 quantitative image features to build prediction models. The performance of prediction models was assessed by calculating the area under the curve (AUC). The best prediction model yielded AUC (95%CI) = 0.83 (0.68, 0.92) using the Thin-Smooth reconstruction setting. The AUC of models using thick slices was significantly lower than that of thin slices (P < 10^−3^), whereas the impact of reconstruction kernel was not significant. Our study showed that the optimal prediction of EGFR mutational status in early stage LACs was achieved by using thin CT-scan slices, independently of convolution kernels. Results from the prediction model suggest that tumor heterogeneity is associated with EGFR mutation.

## Introduction

Lung cancer is the leading cause of cancer death for men and women in the U.S. and worldwide^1^. Adenocarcinoma is the main histological subtype of non–small cell lung carcinoma. A key mechanism of the tumorigenesis of adenocarcinoma is somatic mutations of the epidermal growth factor receptor (EGFR) gene, which leads to the overexpression of EGFR tyrosine kinase receptor in lung tumor tissue^2^. When ligands bind to the EGFR receptor, the molecule is phosphorylated and activates a downstream signaling pathway that inhibits apoptosis and mediates cancer cell growth, proliferation, and invasion. This creates autocrine and paracrine growth factor loops which promote tumor growth.

The diagnosis of EGFR mutational status on a per patient basis is a key biomarker for defining personalized treatment strategies. The mutation occurs frequently, especially in specific populations including non-smoking females and Asians (the reported frequency of EGFR mutations is 48% in China and 23% in the US^3^), and is therapeutically actionable. Gefitinib is a targeted molecular agent that inhibits EGFR. The EGFR mutation is, therefore, a strong predictor of prolonged progression-free survival and of higher response rate to Gefitinib^4–9^. The efficacy of Gefitinib treatment typically translates into a small magnitude of tumor size decrease and a symptomatic improvement^10,11^.

Individualized cancer treatment strategies are enabled by radiomic signatures associated with a specific gene mutation. Current results suggest that EGFR-mutant tumors have a unique imaging phenotype as compared to ALK-mutant^12–14^ or EGFR-wildtype tumors^12–22^, so that imaging could be used to select patients who will potentially benefit from Gefitinib and direct those without EGFR-mutant tumors to other therapies. Largely due to the lack of reliable software (e.g., tumor segmentation and characterization tools), preliminary data in this area have used qualitative imaging features visually assessed by radiologists which are observer dependent, require training, and are time-consuming to measure. Furthermore, current studies suffer from several limitations. First, most models used qualitative and basic imaging features or a limited set of radiomic features^17,19^. Second, most radiomic studies used retrospective imaging datasets that had heterogeneous imaging settings^16–19^ (e.g., reconstruction kernel, slice thickness) which could affect radiomic features and thus critically alter the accuracy of radiomic signatures^16,18,19,23–26^.

Therefore, in this study, we evaluated whether the optimization of reconstruction settings (i.e. Thin/Thick slice thicknesses, Sharp/Smooth convolution kernels) could allow the construction of a better radiomic signature, derived from a large number of quantitative image features, to predict the EGFR mutation status in primary lung adenocarcinoma (LAC) using standard of care CT imaging.

## Results

### Patient Characteristics

A total of 51 patients were included in this study and scanned with all four CT imaging settings formed by the combinations of two slice thicknesses (1 mm and 5 mm) and two convolution kernels (Sharp and Smooth). Fifty-one primary lung adenocarcinoma tumors (one tumor per patient) were identified for analysis. The patient and tumor characteristics of the 51 patients are provided in Table 1 (The information about plerual invasion and tumor density (solid/partial-solid/GGO) were provided by Y.L., an experienced radiologist with 20-year experience of CT interpretation. The evaluation was done blinded from the tumor mutation status). We can see that female and non-smoker patients were more likely to have EGFR mutant tumors, suggesting that our study cohort was typical of Asian populations^27^. For tumor characteristics, such as size, density and pleural invasion, there were no significant difference between EGFR mutant and Wild Type groups (t-test for continuous data and chi-squared test for categorical data).

**Table 1:** Distribution of patient and tumor characteristics.

	EGFR Mutant	EGFR WildType	P
Number	23	28	
Age (yr, mean ± SD)	58.6 ± 9.42	57.6 ± 9.99	0.714
Gender			<0.01
Male	9	23	
Female	14	5	
Differentiation			<0.01
High/Mid	14	8	
Poor	6	20	
Not-known	3	/	
Stage			0.250
I	4	1	
II	4	5	
III	15	22	
Size	39.45 ± 17.94(mm)	43.33 ± 17.22(mm)	0.436
N-Staging			0.252
0	11	8	
1	1	2	
2	6	11	
3	5	7	
Density*			
Solid	17	16	0.185
Part-Solid	5	11	
GGO	1	1	
Pleural invasion			
Yes	11	14	0.899
No	12	14	
Smoking status			
Non-smoker	17	10	<0.01
Smoker	6	18	

^*^The definition of tumor density is, solid - nodule has homogenous soft-tissue attenuation; partial-solid -nodule consists of both ground glass and solid soft-tissue attenuation components; GGO - nodule manifests as hazy increased attenuation in the lung that does not obliterate the bronchial and vascular margin.

### Reproducible Features

For each lesion in each image group, 1,695 quantitative image features were extracted. By employing the same-day repeat CT dataset^25^, 954, 1,182, 812, and 964 features were identified as reproducible features for the four image groups (Thin-Shp, Thin-Smo, Thick-Shp and Thick-Smo), respectively. In the coarse feature selection, a concordance correlation coefficient (CCC) threshold of 0.8 was applied to generate a compact list of candidate features, removing as redundant all features with correlation greater than 0.8. After applying the correlation threshold, the compact feature list contained 104, 98, 92, and 86 candidate features for the four image groups, respectively. In each compact feature list, only the top ten features were retained for the subsequent fine feature selection and model building.

### Optimal EGFR Prediction Models at Different Imaging Settings

**Figure 1:**
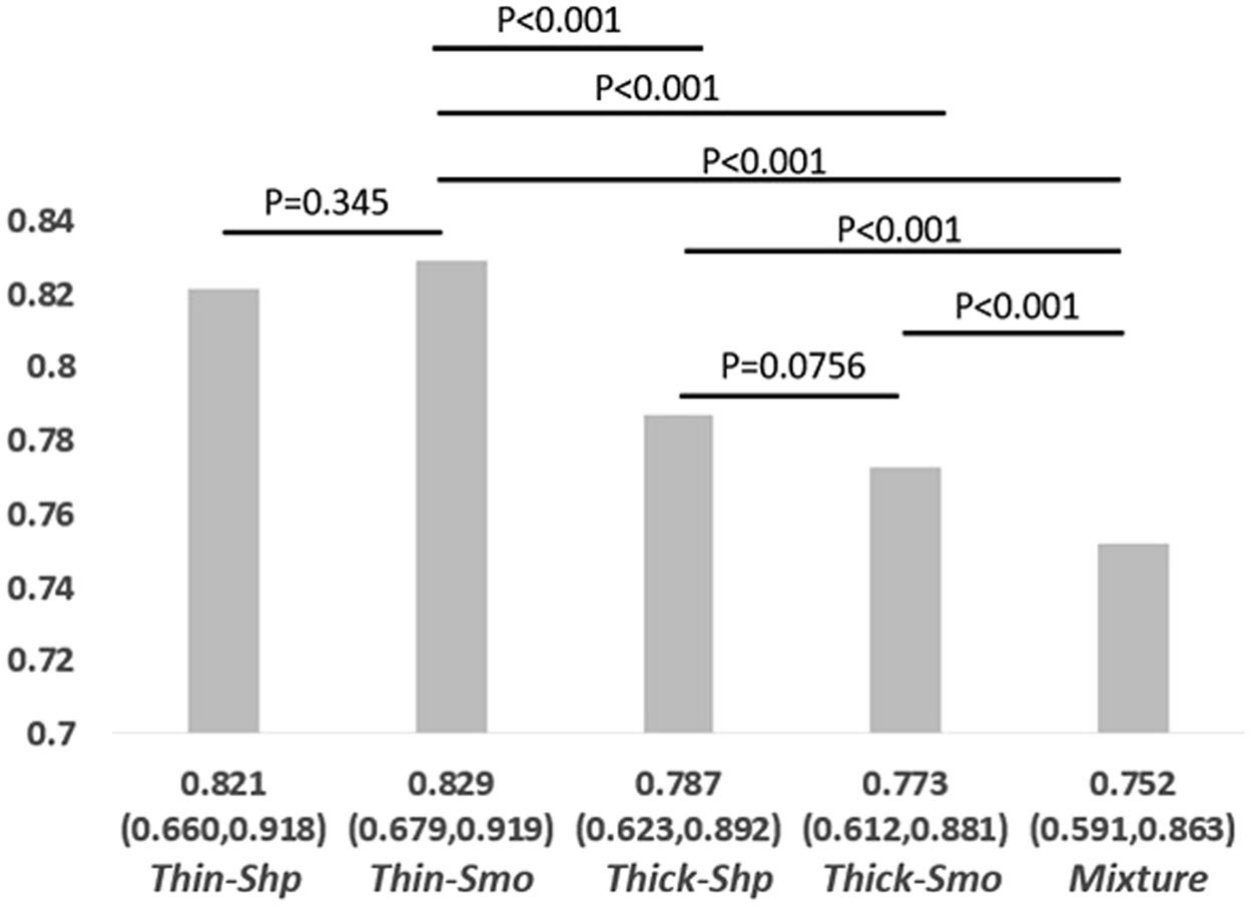
Performances of the optimal models built on different image groups (Thin-Shp, Thin-Smo, Thick-Shp, Thicl-Smo and Mixture).

The purpose of our study was to compare prediction models built upon images grouped according to the different acquisition parameters. Thus, we identified four optimal prediction models for the four imaging setting groups (Thin-Shp, Thin-Smo, Thick-Shp and Thick-Smo). In addition, we constructed a ‘mixture’ group of images for the comparison of prediction models built on homogenous image series vs. those built on heterogeneous acquisition parameters. In the mixture group, the image series for each patient was randomly collected from one of the four image groups and the image features were those reproducible across all the four image settings. For the sake of randomness, we studied ten random ‘mixture’ groups. For the ‘mixture’ model, the performance was the average of the ten randomly constructed models. The performance of the optimal model for each image groups is presented in Fig. 1. The features selected for building those optimal modes are presented in Table 2.

**Table 2:** Selected features for building the optimal models.

Thin-Shp	Thin-Smo	Thick-Shp	Thick-Smo
LoG_Entropy_Sigma2.5_2D	LoG_Entropy_Sigma2.5_2D	GLCM_ASM_2D	Laplacian_Entropy_3D
LoG_Z_Uniformity_Sigma2.5_2D	LoG_Z_Uniformity_Sigma2.5_2D	GTDM_Contrast_2D	Sigmoid_Slope_Std_3D
GTDM_Coarseness_25D	Volume_3D	Sigmoid_Kurtosis_3D	GLCM_Homogeneity_2D
Gabor_dir135_2D	Shape_SI9_3D		Sigmoid_Amplitude_3D
	Gabor_dir45_3D		Laws_8_3D

As shown in Fig. 1, Thin-Smo and Thin-Shp were identified as the best imaging settings to build EGFR prediction models, with the Thin-Smo model (AUC = 0.83) performing slightly better than the Thin-Shp model (AUC = 0.82) (P = 0.345). Thick-Smo and Thick-Shp were identified as the worst imaging settings to build EGFR prediction models, with the Thick-Smo model (AUC = 0.77) slightly worse than the Thick-Shp model (AUC = 0.79) (P = 0.0756). There were statistically significant differences (P < 0.001) between Thin and Thick models. Compared to the four homogenous imaging settings, the Mixture setting group was identified as the worst image group to build EGFR prediction model, significantly worse than the other four settings (P < 0.001). Moreover, the numbers of support vectors for the four optimal prediction SVM models, one for each of the four imaging settings (Thin-Shp, Thin-Smo, Thick-Shp and Thick-Smo), were 10, 12, 16 and 16, respectively, *i.e*., 19.6%, 23.5%, 31.3% and 31.3% of patients were used as the support vectors for the final models, respectively. It is noted that models based on thin-section imaging settings were more generalizable than that based on thick-section imaging settings.

**Figure 2:**
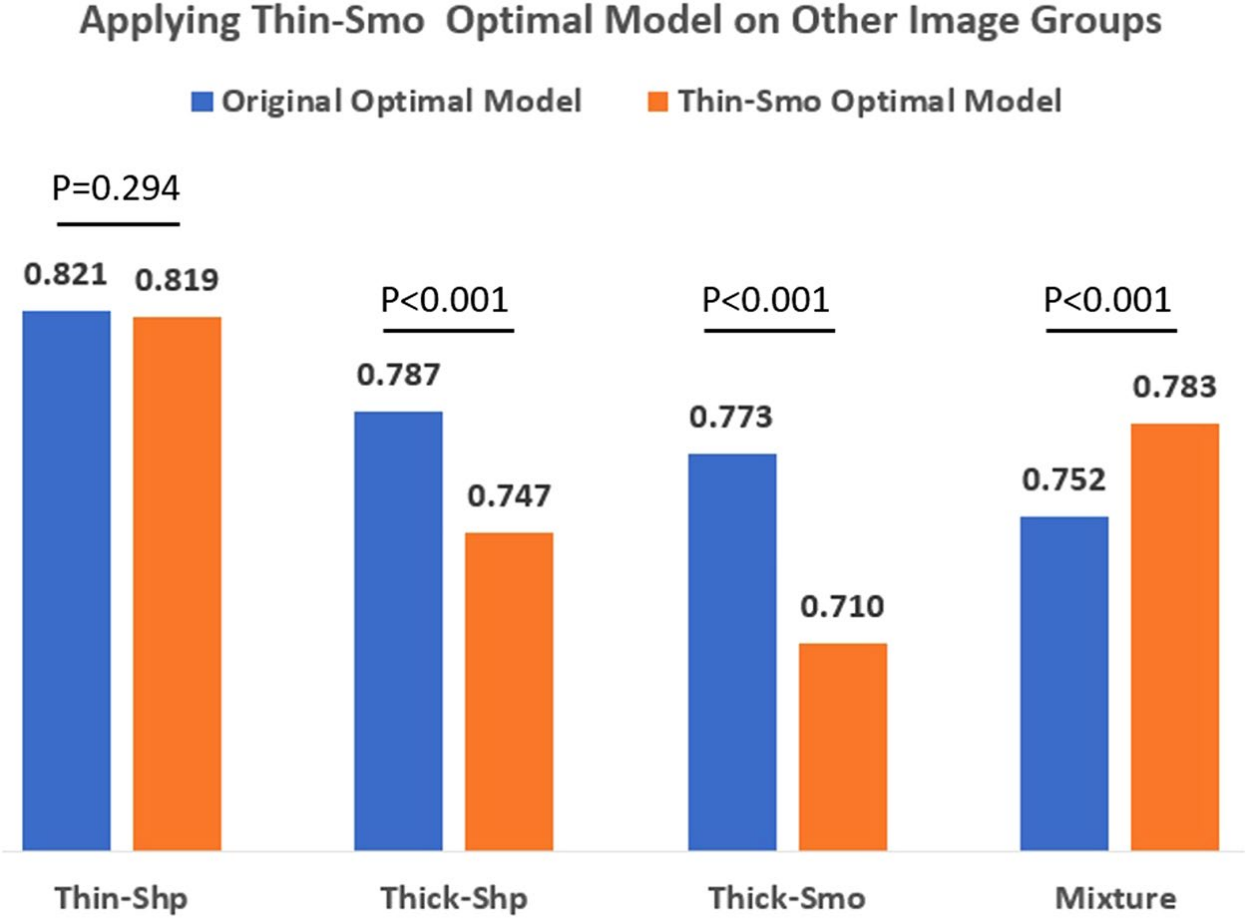
Performances of applying the Thin-Smo optimal model to the other four image groups.

When applying the best model built using the Thin-Smo imaging setting to the other four imaging setting groups, the Thin-Shp group’s performance stayed almost unchanged. The two Thick groups’ performances dropped, but the Mixture group’s performance increased (Fig. 2).

**Figure 3:**
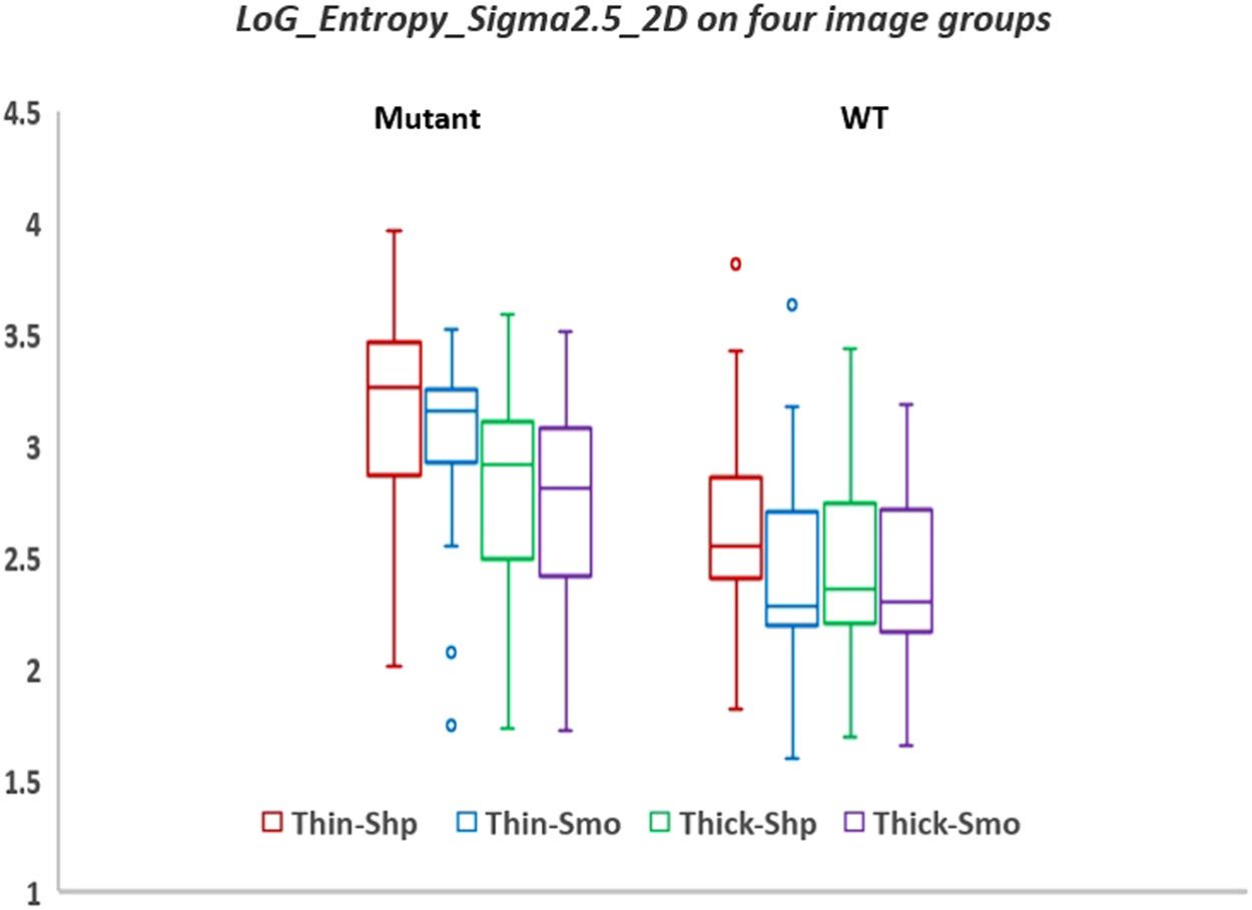
Feature value distributions of Laplacian of Gaussian-Entropy at different imaging setting groups.

It is noticeable that the top two features selected to build the Thin-Smo and Thin-Shp models were the same, LoG_Entropy_Sigma2.5_2D and LoG_Z_Uniformity_Sigma2.5_2D. The higher the ‘LoG_Entropy_Sigma2.5_2D’ value, the more heterogeneous the lesion. As shown in Fig. 3, the median values of ‘LoG_Entropy_Sigma2.5_2D’ on EGFR mutant lesions were larger than those on EGFR wild-type lesions in all four imaging setting groups. Among the four groups, median values of ‘LoG_Entropy_Sigma2.5_2D’ on EGFR mutant lesions decreased from Thin-Shp to Thick-Smo groups, but remained relatively stable on EGFR wild-type lesions.

## Discussion

In this study, we demonstrated that the optimal selection of reconstruction parameters on CT-scan could enhance the predictive value of a radiomics signature to identify EGFR mutation status in early-stage lung adenocarcinoma. We also found that heterogeneous tumors were more likely to harbor the EGFR mutation. Our results showed that thin slices are the optimal reconstruction setting among the four most commonly used CT imaging parameters, as using thick slices significantly altered the predictive value of radiomics (P < 10^−3^).

Our study demonstrated that the thin slices yielded AUCs of 0.82 and 0.83 for the prediction of EGFR mutation using sharp and smooth convolution kernels respectively. As a comparison, thick slices yielded AUCs of 0.79 and 0.77 using sharp and smooth convolution kernels respectively. The impact of convolution kernel was not found to be significant. This may be because the two most significant features selected for the thin-smo and thin-shp image series, Laplacian of Gaussian Entropy and Laplacian of Gaussian Uniformity, were computed from the images pre-processed by a smoothing filter (Gaussian-filter with a larger-sized filter length). This preprocessing procedure reduced the differences between smooth and sharp images. On a broader perspective, this report further demonstrates the importance of rigorous image acquisitions previously indicated by our group (i.e. impact of reconstruction settings^23^, interobserver variability^28^, and acquisition protocols^29^). Interestingly, a previous study reported that non-contrast, thin-slice, and standard convolution kernel-based CT in solitary pulmonary nodule was more informative and increased the diagnostic performance of a radiomics signature^25^.

**Figure 4:**
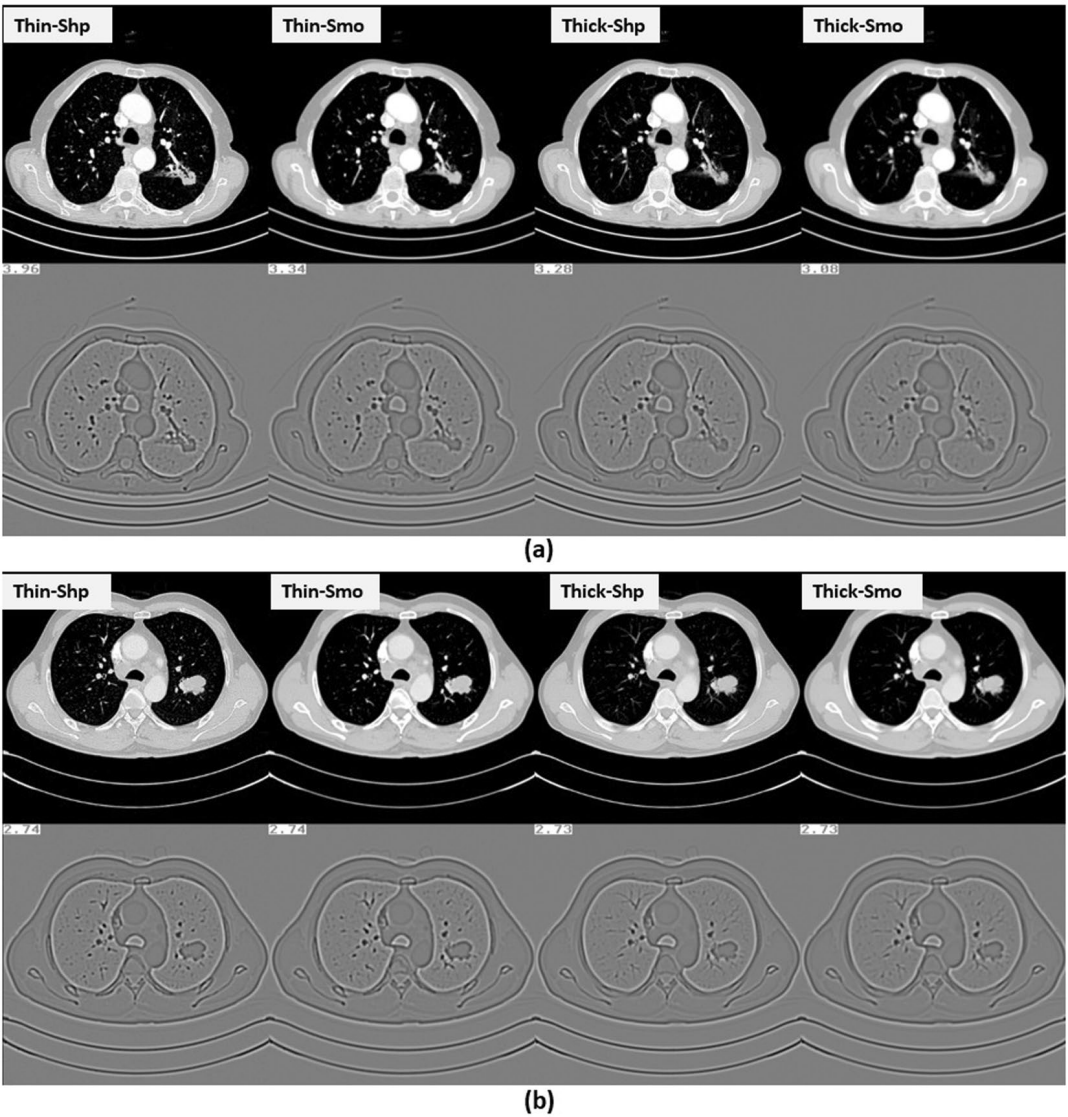
(**a**) An EGFR mutant case. (**b**) An EGFR wild-type case. The four setting images of the two cases were shown from left to right. Images shown at the second row of the sub-pictures were images processed by the Laplacian of Gaussian filter. The values of Laplacian of Gaussian-Entropy were shown at the top-left of the processed images.

Laplacian of Gaussian entropy was a key feature selected in three out of four reconstruction settings. The Laplacian of Gaussian will smooth the image, which might explain why this feature was selected using both sharp and smooth reconstruction settings. This feature captures a heterogeneity pattern in pixel spatial distribution and, interestingly, has been previously found to be associated with tumor phenotype^30^, tumor gene expression, tumor metabolism, tumor stage^31,32^, patient prognosis^33–36^, and treatment response. Laplacian of Gaussian entropy may be considered as a tumor-specific imaging biomarker that is also a function of the primary tumor type, the size of the tumor, and the metastatic site^30^. In this work we show that the difference in this biomarker between EGFR-wildtype and EGFR-mutant is influenced by the reconstruction settings, increasing when using Thin-Sharp reconstruction setting and decreasing when Thin-Smooth, Thick-Sharp and Thick-Smooth reconstruction settings are used. Two example cases, one EGFR mutant and one EGFR wild-type, were presented in Fig. 4.

Our study reported that EGFR tumors are heterogeneous. This is in line with the current literature indicating that EGFR-mutant tumors have a unique imaging phenotype^12–22^, with differences in ground glass opacity, tumor size, pleural retraction and air bronchogram the most frequently reported imaging features. Other EGFR mutant-related features were reported anecdotally such as tumor shape, heterogeneous enhancement, calcification, peripheral fibrosis/emphysema, border definition, spiculation, pleural attachment/effusion, tumor location, nodules in primary tumor lobe, nodules in non-tumor lobes, N-stage, and M-stage^12–22^. Consequently, our study provides an external validation using quantitative image features that enhance the knowledge about the imaging phenotype associated with EGFR mutation in LACs. The independent validation of those EGFR mutant-related imaging features in our series is of crucial significance since type I errors and over-fitting is expected in radiomics studies.

More importantly, we validated our optimal radiomics-EGFR signature (AUC = 0.83) using both homogeneous and heterogeneous CT acquisition settings, a wide range of imaging features (n = 1695), and a machine learning approach. The improvement of the accuracy of the radiomics model in our series is of interest since most previous models were built using basic imaging features, mostly qualitative rather than radiomic per se^12–15,18–22,37^. Prior radiomics studies used a limited number of imaging features (183^16^, 5^17^, 299^18^, 30^19^) compared to the new imaging features implemented in our study. The AUC of previous models based on radiomic features were only 0.67^16^ and 0.71^18^, outperformed by our model (AUC = 0.83). Our study data included patients from a single Chinese institution, allowing them to be benchmarked against previous models which were also designed at a single center institution, mostly in Asia (China, Korea)^12–14,16–22^.

We believe that these results could have major applications since CT-scans guide decision making throughout the course of NSCLC, including screening^38–40^, characterization of lung nodules, TNM staging, biopsy guiding, radiation treatment planning, and response assessment. The outcome of TNM staging is defined by quantitative imaging metrics such as tumor size^41–43^ or binary metrics derived from medical images (such as the involvement of the main bronchus or the presence of atelectasis, pneumonitis, or a diaphragm invasion)^44–46^. Furthermore, guiding personalized treatment by imaging biomarkers offers the prospect of a “virtual biopsy”, which is attractive because conventional biopsies are limited to the sampling site and have a low negative predictive value (68%) and a significant false negative rate (9%)^47^, especially in the case of a large lesion and a sub-solid nodule^48^. Additionally, CT-guided lung biopsies are associated with complications such as pneumothorax and parenchymal hemorrhage^49,50^.

One limitation of our model is that it was built in early stage lung adenocarcinoma, for which treatment is surgery and external beam radiation therapy rather than EGFR inhibitor. However, the definition of the radiomics signature in this model offers several advantages. First, because all patients included in our series had surgery, our reference standard for the identification of EGFR status is robust compared to the determination of EGFR mutational status using biopsies, which suffer from sampling bias. Second, the contours of early stage lung cancers are well defined compared to invasive and/or infiltrative advanced stage lung cancer, in which the determination of the border of the tumor is challenging due to atelectasia, pleuresia, and invasion of other structures by cancer. Third, because biopsies are not always performed in early stage treatment prior to external beam radiation therapies, this is a case in which virtual biopsy through imaging biomarkers is most likely to be useful. Another limitation of this study is the small number of patients, i.e., only 51 patients met the data inclusion criteria. We plan to continue the data collection, from multiple institutions, to validate our findings in the future.

We concluded that the optimal reconstruction setting on CT-scan to predict the presence of EGFR mutations in early stage LAC is thin slices. This could provide a noninvasive method to predict the genetic characteristics of LACs and help personalize patients care.

## Method

### Patients and Image Acquisition

Patient data were retrospectively collected from the Second Xiangya Hospital of Central South University, China. For retrospective study, the institutional review board approved the study before its commencement and waived the requirement for informed consent. Also, all experiments were performed in accordance with relevant guidelines and regulations of the institution. The primary patient cohort in this paper was collected by searching the institutional database for consecutive inpatients who met the following criteria: (1) underwent molecular examination from May 2014 to Dec 2016; (2) had complete histological and clinical information; and (3) were diagnosed for primary Stage I-III lung adenocarcinoma by surgical resection (the 8th Edition of TNM in Lung Cancer). In total, 355 patients were collected, and 315 of them had complete histological and clinical information. Among the 315 patients, 74 patients were diagnosed for primary lung adenocarcinoma at Stage I-III.

Molecular examination was performed on all of these 74 patients using tumor specimens from surgical resection. EGFR wild-type and mutant status was determined by an amplification refractory mutation system real-time technology using Human EGFR Gene Mutations Fluorescence Polymerase Chain Reaction (PCR) Diagnostic Kit (Amoy Diagnostics Co., Ltd, Xiamen, China).

CT images of the 74 patients were searched approximately one month before their surgery. For each patient, multiple imaging series of CT data were collected. For the contrast-enhanced CT, the IV contrast was injected at a rate of 2.5 mL/sec via a pump injector, and the total amount of IV contrast were 60~70 ml. The scanning on thorax began on 30-second delay of the contrast injection. Imaging protocols used to scan the patients are provided in Table 3. Raw data of each patient’s CT scan were reconstructed into four image series: the combinations of two slice thicknesses (Thin: 1 mm; Thick: 5 mm) and two convolution kernels (Sharp: B70f / B70s; Smooth: B30f/B31f/B31s). That is, each patient had four groups of images: 1) Thin-Shp, 2) Thin-Smo, 3) Thick-Shp, and 4) Thick-Smo. The four different image groups differ on levels of image spatial resolution and noise, e.g. Thin-Shp resulted in images with high spatial frequencies and noise preserved, while Thick-Smo resulted in images with low spatial frequencies and noise decreased. As can be seen in Table 3, since all the four image groups were reconstructed by using the exactly same raw scanning data, there was no bias on the comparison among the four groups. Finally, 51 patients having all four imaging settings were used as the study cohort in this paper.

**Table 3:** Distributions of CT scanners and scanning parameters in EGFR mutant and Wild Type groupes.

Image Group	Thin-Shp	Thin-Smo	Thick-Shp	Thick-Smo
Slice Thickness (mm)	1	1	5	5
Convolution Kernels	B70f / B70s	B30f/B31f/B31s	B70f/B70s	B30f/B31f/B31s
	**EGFR Mutant**		**EGFR Wild Type**	**p**
Manufacturer SIEMENS	23		28	/
Scan Model				0.396
Perspective	12		10	
Sensation 64	5		6	
SOMATOM Definition AS	2		1	
SOMATOM Definition Flash	4		9	
SOMATOM Force	0		2	
KVP				0.319
90	0		2	
110	11		10	
120	11		16	
130	1		0	
Exposure (mean ± SD mAs)	123 ± 60		99 ± 55	0.143
Pixel Spacing (mean ± SD mm)	0.655 ± 0.053		0.630 ± 0.055	0.107
Pitch (mean ± SD mm)	1.31 ± 0.56		1.11 ± 0.21	0.153
Contrast Agent				0.753
APPLIED	19		21	
Non-APPLIED	4		7	

### Tumor Segmentation

Tumor segmentation is a procedure to define the lesion area from which radiomic features will be extracted. In our study, 51 lesions (one per patient) were segmented out from their surrounding background by using a semi-automated algorithm^51^ based on watershed and active contours image processing techniques. The semi-automated segmentation was performed by an experienced radiologist (YL with 20 year experience of CT interpretation) on all four image groups. For the sake of consistency, tumor segmentation was first performed on the Thin-Shp group, and then duplicated to the other three image groups. During the duplication, slice re-sampling was used to guarantee the same voxel-resolution between two image series. The radiologist was allowed to edit the duplicated contours if slice re-sampling caused pixel shifting on images.

### Feature Extraction

For each lesion in each image group, totally 1,695 well-defined quantitative image features were extracted by using an in-house feature extraction algorithm implemented in Matlab 2016b (Mathworks, Natick, USA). The 1,695 extracted features are able to characterize tumor phenotypes in terms of size (e.g., largest diameter, volume), shape (e.g., roundness, compactness), sharpness (e.g., Sigmoid slope), texture patterns (e.g., tumor heterogeneity quantified by Laplacian of Gaussian image filter, Gray-Level Co-occurrence Matrix). The 1,695 features represented an expansion of the set of 89 imaging features used in our previous work^23^, achieved through increasing the scales of feature parameters. For instance, four scales of Laplacian of Gaussian filter, 0, 0.5, 1.5, and 2.5 sigma, were used in this study.

### Reproducibility Analysis

A previously collected NSCLC test-retest dataset^52^ was used to assess the reproducibility of the extracted quantitative image features. This test-retest dataset was a CT imaging dataset consisting of 32 NSCLC patients who underwent two repeat CT scans within 15 minutes. The 32 CT scans were reconstructed into six imaging setting groups, four of which were similar to the four reconstruction parameters used in our study. For each image group of the 32 patients, 1,695 radiomics features were extracted from each lesion on both test and re-test scans using the same method presented above. Concordance correlation coefficient (CCC)^53^ was used to evaluate the reproducibility of features for each image group. Features with a CCC larger than 0.9 were included for the subsequent analyses.

### Model Building

In our study, a ‘coarse’ to ‘fine’ strategy was employed to select informative and non-redundant candidate features from the large feature pool consisting of over a thousand features. The coarse selection was fast but only based on the properties of individual features, while the fine selection was time-consuming but based on the combination effect of multiple features.

The coarse selection included two steps, redundancy removal and feature ranking. In the step of redundancy removal, features with high correlation were regarded as redundant features and thus excluded from the following analysis. The procedure included first calculating correlation between features, then organizing all features into a hierarchical clustering tree according to their mutual correlations, and finally setting a correlation threshold to separate all features into a series of redundant groups (i.e. when setting correlation threshold as 0.5, it meant all candidate features are clustered into a series of redundant groups, within which correlation of all feature exceed 0.5). For each redundant group, feature ranking algorithms were applied to rank those correlated features, and only the top-ranked feature was selected for the following analysis. In our study, six feature ranking algorithms were employed, i.e., RELIEF^54^, Chi-square score, Minimum redundancy maximum relevance^55^, T-test score, Wilcoxon score, and Univariance accuracy. Through coarse selection, we created six compact candidate feature lists. Top ten features in each candidate feature list were used for the following analysis.

Fine selection was then applied to determine optimal features to be used to construct EGFR prediction modules. In this step, ‘forward search’ was adopted to evaluate features sequentially. Forward search initiated on an empty set and selected a feature if and only if the addition of the feature could increase the performance of prediction model. The procedure of forward search was repeated until all the candidate features in the compact candidate feature lists were evaluated. The Support Vector Machine algorithm^56^ was used to construct models. As there were six feature lists, a total of six candidate prediction models were generated. Among the six prediction models, the model that achieved the highest performance was selected as the final optimal model for each imaging group.

In the implementation, all the algorithms were coded or download as packages on the Matlab 2016b (Mathworks, Natick, USA) platform. Parameters involved were all used default settings except the Box-Constraint^56^ for the SVM algorithm. SVM algorithm is a machine-learning algorithm that performs classification by finding the hyperplane that maximizes the margin between two classes defined by the so-called support vectors, the percentage of the patient sample set^56^. Theoretically, SVM algorithm can fit any distribution of patients by using support vectors. However, the more the support vectors are used, the higher probability that the SVM model is overfitting. Therefore, SVM algorithm introduces the Box-Constraint, a parameter that controls the maximum penalty imposed on margin-violating support vectors, to help to prevent overfitting, *i.e*., if Box-Constraint increases, then fewer support vectors will be used by the model. In our study, based on our previous experience, we empirically set the Box-Constraint = 100, one hundred times of the default Box-Constraint = 1 in Matlab, to prevent overfitting.

### Performance Evaluation

The performance of candidate prediction model was evaluated in terms of AUC (i.e. the area under the curve of receiver operating characteristic curve^57^). Due to the limited number of patients in the study cohort, we used three-fold cross-validation to estimate the performance of models instead of separating the study cohort into training and testing subsets. In the three-fold cross-validation, original data were randomly partitioned into three groups. When one group was used for testing, then the other groups was retained for training. The training-testing procedures were repeated three times, until each sample in the data set was assigned a prediction score. The final AUC as well as its confidence interval (95%) were estimated based on the prediction score by using bootstrapping (1000 times)^58^. Also, a bootstrap-based approach presented in the literature^59^ was used to compare two models in terms of p-value.

## Data Availability

The datasets generated and analyzed during the current study are available from the corresponding author.
